# Exploring the path of persisting dysfunctional expectations—Development of the immunization scale IMS

**DOI:** 10.3389/fpsyg.2022.1033078

**Published:** 2022-12-08

**Authors:** Anne-Catherine I. Ewen, Winfried Rief, Marcel Wilhelm

**Affiliations:** Department of Clinical Psychology and Psychotherapy, Philipps-University of Marburg, Marburg, Germany

**Keywords:** expectations, cognitive immunization, self-rating questionnaire, psychopathology, ViolEx model, immunization scale IMS

## Abstract

**Objectives:**

Persistent dysfunctional expectations seem to be core features of mental disorders. The aim of this study was to develop a questionnaire that assesses mechanisms responsible for the consistency of dysfunctional expectations. Processes *before* (i.e., assimilation) and *after* (i.e., immunization) expectation-violating experiences have been considered.

**Design:**

The Immunization Scale (IMS) is constructed and validated with the help of an explorative (EFA) and confirmatory factor analysis (CFA) in two conducted studies.

**Materials and methods:**

For the first study, the initially formulated 75-item version was completed online by 230 (range 18–69) participants from a convenience sample. For the second study, 299 (range 18–62) participants completed the reduced scale at the first measurement point, 75 participants thereof also 1 month later. For validity and reliability analyses, participants in both studies provided demographic information, the Beck Depression Inventory (BDI-II), the Depressive Expectation Scale (DES), the Beck Anxiety Inventory (BAI), and the German version of the Acceptance and Action Questionnaire (FAH-II).

**Results:**

The initial 75 items were reduced to 23 items. The EFA revealed three main factors, namely, negative expectations, assimilation, and cognitive immunization. The three-factor structure could be confirmed in study 2 by the CFA. Reliability measures showed an excellent internal consistency for the entire IMS. A very good test–retest reliability was found. Significant correlations resulted between the IMS and DES, BDI-II, BAI, and FAH-II, the highest for DES and FAH-II.

**Conclusion:**

Psychometric properties of the IMS are promising. Future studies should verify the reliability and validity measures in other population samples. The IMS can be very useful in expectation research, especially in the examination of expectation-focused therapy.

## Theoretical background

The concept of expectations playss an important role in explaining human functioning. Expectations have been part of theoretical frameworks in psychology for decades. In social psychology, self-fulfilling prophecy and the Pygmalion effect (Rosenthal and Jacobson, 1968) are typical theoretical examples representing the power of expectations. Furthermore, expectancy value theories summarize different decision and action theories with the aim to explain human behavior, for example, Atkinson’s theory of achievement motivation (Atkinson and Feather, 1966), the Rubicon model of action phases (Heckhausen and Gollwitzer, 1987), or the prospect theory describing choice behavior in economic and decision psychology (Kahneman and Tversky, 1979).

In clinical psychology, expectations gained explicit relevance as the most important mechanism of the placebo effect (Imel et al., 2008; Wampold and Imel, 2015; Kirsch et al., 2016). As early as 1961, Jerome D. Frank postulated that psychotherapy works mainly by building positive expectations for improvement. Greenberg et al. (2006) also argued that most psychotherapies inevitably go hand in hand with the change and revision of patients’ expectations. Accordingly, research integrated different forms of expectations as predictors of therapy outcomes (Constantino et al., 2012). Depressed patients’ expectations of outcome are associated with therapeutic alliance and alliance expectations (Barber et al., 2014). Meta-analytic evidence showed that patients’ presurgical expectations determine postsurgical outcomes and postoperative quality of life (Auer et al., 2016). In psychotherapy, the effect size of early treatment outcome expectations on patients’ posttreatment outcome seems to be small but significant (Constantino et al., 2018). Therefore, patients’ expectations should be measured by the psychotherapist in order to predict and possibly improve therapy outcomes.

Thus, recent research assigns a pivotal role to expectations in psychotherapy, defining them as “core features” of mental disorders (Rief et al., 2015). Not only do many patients have overly negative expectations prior to therapy, many also fail to update those expectations following expectation-disconfirming experiences. Pinquart et al. (2021a) compared different models dealing with expectations concluding the following three important process mechanisms: (1) Expectations can be changed; (2) expectations can be maintained by retroactively minimizing the importance of expectation-disconfirming evidence; and (3) expectations can be maintained by selectively searching for or producing expectation-confirming evidence. One proposed model explaining the persistence or changeability of expectations is the ViolEx model (Rief et al., 2015). Different reactional information-processing mechanisms to an expectation-violating experience are proposed: assimilation and immunization (Gollwitzer et al., 2018; Pinquart et al., 2021b). Assimilation describes the concept of searching or producing expectation-confirming information (i.e., avoidant behavior). It consists of two mechanisms: (1) defining the avoidance of any possible expectation inconsistent experiences and (2) defining the active contribution seeking expectation-confirming information (i.e., self-fulfilling prophecy). Immunization describes the concept of reappraising inconsistent evidence in a way that it is no longer disconfirming the expectation. As an example, a patient may expect that the therapist will not take him seriously. The empathic behavior of the therapist can be seen as an expectation violation, whereas the cognitive immunization “it is just his job, he is not really interested in my problems” will inhibit the expectation change. If the patient would avoid going to a therapist, this behavior would be described as an assimilation process in the sense of the ViolEx model.

In anxiety disorders, situation-specific, dysfunctional expectations are already successfully targeted by performing exposure therapy with cognitive elements, such as seeking situations most likely violating the specific expectation, leading to a faster and efficient change in the dysfunctional expectation (Craske et al., 2014). This directly targets the problematic behavior (i.e., avoidance) that occurs *before* an expectation-violating situation. Patients with anxiety disorders are often avoiding situations presumed to be dangerous, which leads to a non-experience of expectation violation and makes it impossible to update the expectation of danger (Marks, 1979; Myers and Davis, 2007; Pittig et al., 2020). Patients with depression also tend to have a higher amount of dysfunctional negative expectations toward future events (Kube et al., 2017) and fail to update their expectations after an expectation violation, suggesting the involvement of immunization processes (Kube et al., 2019c). These immunization processes cause experienced expectation violations to be (re)interpreted as exceptions instead of a new experience (Rief et al., 2015). Thus, the flexible formation of expectations and their adaptation to the environment seem to be disturbed. Liknaitzky et al. (2017, 2018) postulate cognitive rigidity, probably a consequence of high immunization processes, as a crucial obstacle in changing interpretations, beliefs, and expectations in people with depression.

Some interventions addressing dysfunctional expectations and cognitive immunization processes have already been developed (Rief and Glombiewski, 2016; Kube et al., 2019a,b). However, as a validated instrument to measure the patient’s immunization level is lacking, cognitive immunization being responsible for the patient’s rigid expectations is a presumption. The goal of this study was to develop and validate a questionnaire operationalizing the main mechanisms presumably responsible for persisting dysfunctional expectations. In the first step (*study 1*), a questionnaire based on the theoretical background is constructed and a factor structure is established. In the following step (*study 2*), the shortened questionnaire is confirmed in an independent sample.

## Materials and methods

### Ethics

The local ethics committee of the Department of Psychology, Philipps-University Marburg, approved the study (reference number 2020-31k).

### Procedure, concept definition, and scale development

For elaborated scale development, an extensive literature review on the two main constructs, namely, *assimilation* and *immunization*, was conducted. This was followed by a discussion with psychologists, psychotherapists, and researchers concerning their understanding of mechanisms leading to the persistence of (dysfunctional) expectations and a lack of expectation adaptation. The questionnaire was designed as a transdiagnostic measure, as assimilation and immunization processes behavior can be found in different kinds of psychopathologies (Kashdan and Rottenberg, 2010).

Consequently, and in line with the ViolEx model, a distinction between mechanisms that occur before an experience of expectation violation and mechanisms after an experience of expectation violation should be considered. In addition to the concept of cognitive immunization (Rief et al., 2015) and assimilation (Gollwitzer et al., 2018), other defined constructs were taken into account. In another article including the ViolEx model, the concept of behavioral immunization is proposed (Rief and Joormann, 2019). They distinguish between cognitive and behavioral immunization, both leading to the invalidation of a positive expectation-violating experience. As examples for behavioral immunization avoiding expectation-violating situations, selective attention or ignoring contradictory information is mentioned. It is important to note that behavioral immunization includes different mechanisms occurring *before* (e.g., avoiding the situation), *in* (e.g., attentional processes), and *after* (e.g., avoiding a second expectation-violating situation) an expectation violation. The distinction between processes that are solely cognitive or solely behavioral is nearly impossible. Furthermore, the concept of behavioral avoidance, well-known in anxiety disorders as the consequence of cognitive or emotional processes, should be considered as a mechanism *before* an expectation-violating situation. But also here, expectations seem to mediate the link between avoidance and anxiety (Lovibond et al., 2008). In other words, people with anxiety seem to practice avoidance precisely because they anticipate a negative outcome. They avoid a certain situation with the possible occurrence of expectation violation due to disbelief in a positive outcome (e.g., “I am not going to join the party, because I know, it will be terrible”). In the Acceptance and Commitment Therapy literature, this process is called fusion with proper thoughts leading to psychological inflexibility and experiential avoidance (Hayes et al., 2012).

Conceptually, it makes sense to group these processes together as “invalidating the effect of positive experiences” (Rief and Joormann, 2019), whether they occur before, during, or after the expectation violation. As this study aimed to assess different processes leading to the persistence of negative expectations, we distinguished between processes which occur *before* and *after* expectation-violating experiences. For simplicity, all processes involved before an expectation violation will be referred to as assimilation [following Gollwitzer et al. (2018)], while processes involved in maintaining expectations after an expectation violation will be referred to as immunization. The concept of assimilation seems to be more consistently and durably used in the literature. As these processes are mostly unconscious, the focus of the item formulation was based on the behavioral and cognitive outputs of these different processes.

Further constructs such as pessimism (Chang et al., 1994; Gillham et al., 2001; Herzberg et al., 2006), neuroticism (Claridge and Davis, 2001; Ormel et al., 2013), openness for new experiences (Wolfestein and Trull, 1997; Chiappelli et al., 2021), emotion regulation (Joormann and Gotlib, 2010; Berking and Wupperman, 2012; Gross and Jazaier, 2014; Joormann and Stanton, 2016), external or internal control belief (Burger, 1984; Presson and Benassi, 1996), and cognitive and psychological flexibility (Stange et al., 2017; Coto-Lesmes et al., 2020) have been identified to be overlapping with the concept of assimilation and cognitive immunization.

The items were originally formulated in German; a native English speaker translated the questionnaire into English. A five-point Likert scale to rate the items has been chosen from 1 = *Do not agree* to 5 = *Agree*. A higher sum score indicates a higher level of assimilation and immunization behavior.

After a first pretest (*n* = 15), items were optimized for understanding. Pilot recruitment was launched in May 2020 with 95 initially formulated items. A first item analysis and correlation matrix was conducted with a sample size of 139 healthy participants (mean_age_ = 28.14, SD = 10.79; 72% women, 28% men). A good internal consistency of α = 0.88 was reached for the 95-item questionnaire. Qualitative questions were especially taken into account regarding item composition. After the first test sample, 47 out of 95 items were discarded due to bad item discrimination, poor item understanding, and item formulation, whereas another 27 items were added.

The adapted questionnaire resulted in a 75-item scale. Considering the first sample, these items were intended to fit four subscales, namely, *number of negative expectations, general psychological flexibility, avoidant behavior before an experience of possible expectation violation, and cognitive immunization after an experience of expectation violation.* For the final dataset, 230 healthy subjects filled out the questionnaire.

### Participants

Participants for both studies were mainly recruited through mailing lists, social networks (i.e., Facebook, Twitter, Instagram, and LinkedIn), and participant recruitment pages (i.e., SurveyCircle, Thesius). For remuneration, participation in a voucher raffle of four 25 euro coupons redeemable for online media was offered to the participants.

The recruitment for the final sample for study 1 was done between July and September 2020. In total, 366 participants followed the link and agreed to the informed consent, 44 of whom already exited the study immediately after the informed consent process. Of those who continued, 230 participants completed the study.

The recruitment for the second study took place between January and March 2022. In total, 597 participants followed the link (249 interrupted the study directly after confirming the informed consent). Of these, 299 participants completed T1, and 136 participants started T2. Seventy-five participants completed both time points.

### Other measurements

#### Sociodemographics

Participant information included age, sex, native language, nationality, education, current or past mental disorder, and current or past psychotherapy.

#### Depressive symptoms

For the assessment of depressive symptoms, the validated and reliable German version of the Beck Depression Inventory-II (Hautzinger et al., 2009) was used. The inventory consists of 21 depressive symptoms that are rated in severity and presence in the past 2 weeks on a four-point rating scale (0–3). Based on the sum scores, the cut-off values indicate minimal, mild, moderate, and severe depression. The internal consistency of our sample can be considered as excellent, with a Cronbach’s alpha of α = 0.94.

#### Anxiety symptoms

For the assessment of subjective experienced anxiety symptoms, the validated and reliable German version of the Beck Anxiety Inventory (Margraf and Ehlers, 2007) was used. With 21 items, the BAI assesses the presence of different anxiety symptoms during the last week on a four-point rating scale. A categorization into minimal, mild, moderate, and clinically relevant anxiety is defined through sum score cut-off values. In this sample, an excellent internal consistency (α = 0.92) could be reached.

#### Negative expectations

Situation-specific depressive expectations were measured using the German version of the Depressive Expectations Scale (Kube et al., 2017). Four subscales were defined, namely, expectations about social rejection, social support, emotion regulation (i.e., being helpless in coping with negative mood), and ability to perform (i.e., being helpless in coping with performance-related situations). In this sample, the 25-item self-report measure showed a good internal consistency (α = 0.83).

#### Psychological flexibility

The German version of the Acceptance and Action Questionnaire II (Hoyer and Gloster, 2013) is a validated and reliable self-reported seven-item scale measuring psychological flexibility and experiential avoidance. A higher sum score represents higher inflexibility. In this sample, a good internal consistency could be reached (α = 0.87).

### Statistical analyses

All analyses are conducted using RStudio version 1.2.5042 (RStudio, 2009–2020). First, the IMS was checked for outliers. In study 1, six outliers and in study 2, five outliers could be identified, showing critical values in calculated boxplots (1,5*IQR), and 10 outliers were identified by the Mahalanobis distance. The authors decided to first include the outliers in the calculations.

For study 1, comprehensive item analysis was calculated with the first and second samples, including item difficulty, item total correlations for item discrimination, and Cronbach’s alpha as a reliability measure. Furthermore, parallel analysis appropriate for Likert-type data by using polychoric correlation matrices (Weng and Cheng, 2005) with 100 simulations was calculated to determine the number of factors for the initial factor analysis (EFA). An EFA with diagonally weighted least squares estimation and varimax rotation was conducted with the included items fulfilling the inclusion criteria of the item analysis. The Kaiser–Meyer–Olkin criterion and Bartlett’s test of sphericity were calculated to guarantee the suitability of our data for structure detection. Furthermore, items with factor loadings > 0.30 can be attributed to the corresponding factor (Costello and Osborne, 2005). Pairwise correlations were calculated between the sum scores of the IMS, its subscales, BDI-II, BAI, FAH-II, and DES for reliability and validity analyses. An alpha error level was set at 5%.

In the second study, the lavaan package (Rosseel et al., 2017) was used to calculate the confirmatory factor analysis (CFA). As before, the factor analysis was followed by correlations and *t*-tests to evaluate a test–retest reliability and associations between the IMS and the other validation measurements. An alpha error level was set at 5%. No missing values had to be dealt with.

### Data availability

The data that support the findings of this study are available from the corresponding author upon reasonable request.

## Results

### Study 1

#### Sample characteristics

The data of 230 participants were included in our analyses. The mean age of the sample was 30.08 years (*SD* = 11.49), and 77% of the subjects were female. German was the native language of 91, and 83% had German nationality. Higher education (university degree) was indicated in 42% of the participants.

Means and standard deviations for all questionnaires are reported in Table 1. The mean sum score of the BDI-II was 10.93 (*SD* = 10.70), while 71.74% of the participants reached a BDI-II sum score ≤ 13, indicating the presence of no to minimal depressive symptoms, 12.18% showed mild, 9.57% showed moderate, and 6.52% showed severe depressive symptoms (Hautzinger et al., 2009). The mean sum score of the BAI was 10.40 (*SD* = 9.53). 48.26% of the participants had no to minimal anxiety level, 28.26% showed mild, 15.65% showed moderate, and 7.83% showed clinically relevant anxiety symptoms (Margraf and Ehlers, 2007). In the DES questionnaire, a mean sum score of 59.35 (*SD* = 12.02) was found. A mean sum score of 22.33 (*SD* = 10.62) for inflexibility measured with the German version of the AAQ-II (Hoyer and Gloster, 2013) was found. The distributions of the different questionnaires as well as the correlations between them are shown in Figure 2 (pair panels).

**TABLE 1:** Demographics: Mean and standard deviations of different variables involved in study 1 and study 2.

	Total sample study 1 (*n* = 230)	Total sample study 2 (*n* = 299)
Age [M (SD)]	30.08 (11.49)	25.85 (9.52)
Gender [F/M/other]	178/51/1	213/85/1
M_BDI_ [(SD)]	10.93 (10.70)	9.59 (9.26)
M_BDI female_ [(SD)]	10.94 (10.47)	10.38 (9.71)
M_BDI male_ [(SD)]	9.86 (8.98)	7.61 (7.80)
M_BAI_ [(SD)]	10.40 (16.16)	9.88 (9.34)
M_BAI female_ [(SD)]	10.85 (9.76)	11.24 (9.87)
M_BAI male_ [(SD)]	9.04 (8.58)	6.49 (6.85)
M_DES_ [(SD)]	59.35 (12.02)	56.56 (10.72)
M_DES female_ [(SD)]	59.39 (12.05)	57.04 (11.14)
M_DES male_ [(SD)]	59.25 (12.15)	55.15 (9.44)
M_FAH_ [(SD)]	22.33 (10.62)	21.09 (9.33)
M_FAH female_ [(SD)]	23.11 (10.66)	21.81 (9.31)
M_FAH male_ [(SD)]	19.94 (10.10)	19.20 (9.20)

n, sample size; M, mean; SD, standard deviation; F, female; M, male; BDI-II, Beck Depression Inventory; BAI, Beck Anxiety Inventory; DES, Depressive Expectation Scale; FAH-II, German version of the Acceptance and Action Questionnaire; IMS, Immunization Scale.

**FIGURE 1:**
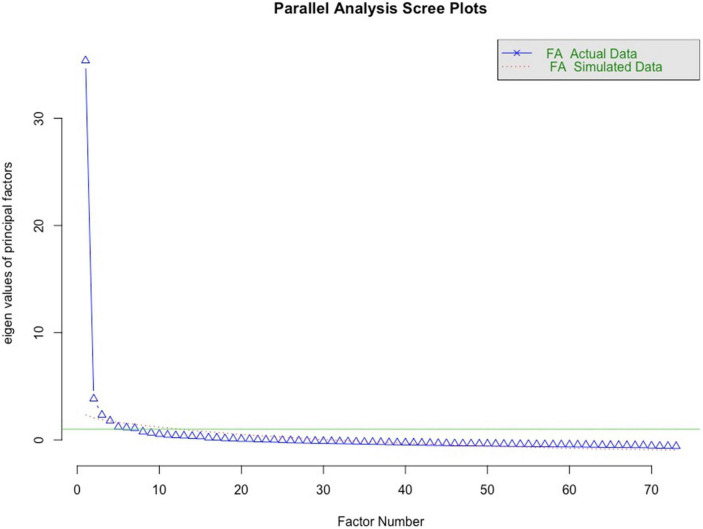
Scree plot suggesting four factors for the 73-item scale.

**FIGURE 2:**
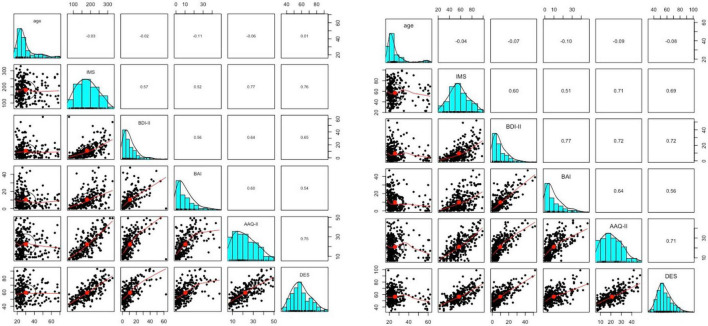
Paired panels indicating the distributions by histograms, the linearity by the scatter plots and bivariate Pearson’s correlations between the different sum scores of the questionnaires and the immunization scale (IMS) for study 1 **(left)** and study 2 **(right)**.

#### Item analysis

All answer options (from 1 to 5) were ticked for each item. Items showing a lower item-total correlation below 0.40 were excluded to guarantee a homogeneous item-pool and a good item-total correlation (Moosbrugger and Kelava, 2012). In this sample, two items showed an item-total correlation below 0.40, whereupon they were excluded resulting in a 73-item scale (for the results of the item analysis see Supplementary marterial A1). Furthermore, the theoretical scale *general psychological flexibility* showed a lot of items with high item-total correlations above 0.70, but lower than 0.80 (range = 0.49–0.76).

#### Exploratory factor analysis

An EFA has been conducted with the 73-item scale to describe the factor structure including the theoretical assumption of the following factors: *quantity of negative expectations, general psychological flexibility, assimilation*, and *cognitive immunization after expectation violation.* Based on the parallel analysis (Figure 1), four factors were considered. The Kaiser–Meyer–Olkin test indicated very good sampling adequacy of 0.95 [range of items: 0.89–0.97]. The Bartlett test showed a heterogeneity of variance [χ2 (72) = 148.04, *p* < 0.0001], indicating a conduction of a factor analysis as reasonable.

A total variance of 53% can be explained by the assumed four factors (for the results of the EFA see Supplementary marterial A2). The theoretical scale *general psychological flexibility* had a high item-total correlation, and accordingly, the factor analysis showed unclear factor attribution of these items. According to these findings, the authors decided to discard these 19 formulated items. All items loading less than 0.3 on a factor were excluded. Every item kept should load at least 0.5 on a specific factor. Every item loading on two factors higher than 0.40 was excluded. Moreover, every item has been checked for redundancy. When items were found to be redundant in the content, the item with the higher and clearer factor loading was chosen. At the end, a 23-item scale resulted in a supposed three-factor structure (see Table 2).

**TABLE 2:** Item loadings of the explorative (EFA) (study 1).

	Factor 1	Factor 2	Factor 3	Factor 4
***Quantity of negative expectations***				
Item 2	0.31	**0.66**	0.13	0.20
Item 3	0.12	**0.65**	0.24	0.16
Item 9	0.35	**0.68**	0.15	0.18
Item 11	0.22	**0.76**	0.12	0.19
Item 12	0.30	**0.57**	0.17	0.11
Item 7	0.29	**0.68**	0.27	0.26
***Assimilation***				
Item 8	0.26	0.14	**0.53**	0.21
Item 10	0.13	0.15	**0.57**	0.24
Item 12	0.13	0.15	**0.54**	0.10
Item 13	0.24	0.28	**0.62**	0.14
Item 14	0.29	0.27	**0.62**	0.22
Item 15	0.33	0.30	**0.55**	0.24
***Cognitive immunization***				
Item 7	**0.60**	0.29	0.11	0.25
Item 8	**0.68**	0.13	0.18	0.28
Item 12	**0.52**	0.22	0.11	0.13
Item 13	**0.67**	0.13	0.21	0.24
Item 19	**0.68**	0.35	0.11	0.12
Item 20	**0.66**	0.26	0.21	0.12
Item 21	**0.72**	0.36	0.00	0.19
Item 23	**0.68**	0.33	0.19	0.00
Item 24	**0.61**	0.22	0.26	0.27
Item 25	**0.71**	0.13	0.17	0.21
Item 26	**0.57**	0.30	0.21	0.00

Highest factor loadings are marked in bold.

#### Reliability and validity analyses

While the 75-item scale showed an excellent internal consistency (Cronbach’s alpha α = 0.98), the 23-item scale is not inferior, also showing an excellent Cronbach’s alpha of α = 0.94. The Cronbach’s alpha of the following factors: *negative expectations* (α = 0.87), *assimilation* (α = 0.85), and *cognitive immunization* (α = 0.93), showed a good to excellent reliability.

The three subscales, although representing three different factors, seem to be highly correlated with one another (*r* = 0.58–0.65). This indicates that individuals with a high amount of negative expectations seem to show a higher level of assimilation and immunization processes. For convergent validity, bivariate associations between the described questionnaires were calculated, whereby depressive symptoms, anxiety symptoms, negative expectations, and experiential avoidance were highly correlated with the sum score of the 23-item IMS (Table 3).

**TABLE 3:** Pearson’s correlations between the sum scores of used questionnaires measuring depressive symptoms, anxiety symptoms, depressive expectations, and psychological inflexibility as well as the sum score of the constructed 23-item and 73-item questionnaire and the three factors of the immunization scale (IMS) negative expectations, assimilation, and cognitive immunization (study 1).

	BDI-II	BAI	DES	FAH-II	73-item IMS	23-item IMS	Factor 1	Factor 2	Factor 3
BDI-II	-	0.56***	0.65***	0.64***	0.57***	0.54***	0.59***	0.39***	0.44***
BAI		-	0.54***	0.60***	0.52***	0.49***	0.56***	0.37***	0.37***
DES			-	0.75***	0.76***	0.73***	0.75***	0.52***	0.62***
FAH-II				-	0.77***	0.74***	0.81***	0.55***	0.59***
73-item IMS					-	0.92***	0.86***	0.78***	0.87***
23-item IMS						-	0.85***	0.80***	0.91***
Factor 1							-	0.59***	0.65***
Factor 2								-	0.58***
Factor 3									-

***p < 0.001. BDI-II, Beck Depression Inventory; BAI, Beck Anxiety Inventory; DES, Depressive Expectation Scale; FAH-II, German version of the Acceptance and Action Questionnaire; IMS, Immunization Scale; factor 1 represents the subscale negative expectations of the IMS, factor 2 assimilation, and factor 3 cognitive immunization.

The sum score of the IMS is not significantly correlated with the age (*r* = −0.04) or education level (*r* = −0.03).

### Study 2

#### Sample characteristics

The data of 299 participants were included in our analyses. The mean age of the sample was 25.85 years (*SD* = 9.72), and 71% of the subjects were female. German was the native language of 94, and 94% had German nationality. Higher education (university degree) was indicated in 24% of the participants.

The different mean and standard deviations in the assessed questionnaires are reported in Table 1. The mean sum score of the BDI-II was 7.61 (*SD* = 7.80), while 73.58% of the participants reached a BDI-II sum score ≤ 13, indicating the presence of none to minimal depressive symptoms, 13.71% showed mild, 7.36% showed moderate, and 5.35% showed severe depressive symptoms (Hautzinger et al., 2009). The mean sum score of the BAI was 9.88 (*SD* = 9.33). 54.18% of the participants had no to minimal anxiety level, 23.08% showed mild, 14.72% showed moderate, and 8.03% showed clinically relevant anxiety symptoms (Margraf and Ehlers, 2007). In the DES questionnaire, a mean sum score of 56.56 (*SD* = 10.72) was found. A mean sum score of 21.09 (*SD* = 9.33) for inflexibility measured with the German version of the AAQ-II (Hoyer and Gloster, 2013) was found (see Figure 2 for pair panels).

#### Confirmatory factor analysis

Results of the CFA based on a three-factor structure suggest a good model fit with *X*^2^ (227) = 193.32, *p* = 0.949, a good comparative fit index of CFI = 1.00, a good normed fit index (NFI) = 0.983, a good Tucker–Lewis index (TLI) = 1.003, and a good root mean square error of approximation (RMSEA) = 0.000 (90% confidence interval = 0.000–0.002).

#### Reliability and validity analyses

The 23-item scale showed an excellent internal consistency with a Cronbach’s alpha of α = 0.93. The Cronbach’s alpha of the following factors: *negative expectations* (α = 0.89), *assimilation* (α = 0.77), and *cognitive immunization* (α = 0.93), showed a good to excellent reliability. For the test–retest reliability, we found a high consistency over time (4 weeks) with a correlation of *r* = 0.87 [*t*(73) = 15.18, *p* < 0.001], suggesting that the IMS reliably measures the underlying construct over time.

Like in study 1, the three subscales, although representing three different factors, seem to be highly correlated with one another (*r* = 0.49–0.56). Bivariate associations between the described questionnaires were calculated, whereby depressive symptoms, anxiety symptoms, negative expectations, and experiential avoidance were highly correlated with the sum score of the 23-item IMS (Table 4). The sum score of the IMS is not significantly correlated with the age (*r* = −0.04) or education level (*r* = −0.03). A significant difference in the IMS sum score between individuals with a current diagnosed mental disorder (*M* = 55.66, *SD* = 15.86) and without (*M* = 68.75, *SD* = 19.35) was found [*t*(31) = 3.46, *p* = 0.002]. The same could be found for a diagnosed mental disorder in the past [*M* = 55.81, *SD* = 15.86; *M* = 62.67, *SD* = 19.37; *t*(31) = 2.23, *p* = 0.03]. Additionally, individuals who reported having received psychotherapy at some point in the past (*M* = 61.91, *SD* = 18.28) showed a significantly higher IMS score than those who had not [*M* = 55.11, *SD* = 15.66; *t*(119) = 2.93, *p* = 0.004]. This difference could not be found for individuals currently in psychotherapy (*M* = 63.52, *SD* = 19.78) vs. not in psychotherapy [*M* = 56.33, *SD* = 16.26; *t*(25) = 1.70, *p* = 0.102].

**TABLE 4:** Pearson’s correlations between the sum scores of used questionnaires measuring depressive symptoms, anxiety symptoms, depressive expectations, and psychological inflexibility as well as the sum score of the immunization scale (IMS) and the three factors of the IMS negative expectations, assimilation, and cognitive immunization (study 2).

	BDI-II	BAI	DES	FAH-II	IMS	Factor 1	Factor 2	Factor 3
BDI-II	-	0.77***	0.72***	0.72***	0.60***	0.66***	0.41***	0.46***
BAI		-	0.56***	0.64***	0.51***	0.59***	0.35***	0.36***
DES			-	0.71***	0.69***	0.67***	0.43***	0.60***
FAH-II				-	0.71***	0.80***	0.47***	0.52***
IMS					-	0.82***	0.75***	0.89***
Factor 1						-	0.55***	0.56***
Factor 2							-	0.49***
Factor 3								-

***p < 0.001. BDI-II, Beck Depression Inventory; BAI, Beck Anxiety Inventory; DES, Depressive Expectation Scale; FAH-II, German version of the Acceptance and Action Questionnaire; IMS, Immunization Scale; factor 1 represents the subscale negative expectations of the IMS, factor 2 assimilation, and factor 3 cognitive immunization.

## Discussion

The IMS is the first self-rating scale measuring assimilation and cognitive immunization as it is defined in the ViolEx model (Rief et al., 2015), which are assumed to be responsible for persisting dysfunctional expectations (validated German version of the IMS can be retrieved from the Supplementary material A3. An English translation of the IMS is also proposed under A3). This article includes psychometric properties and factorial structure of the IMS in a mainly healthy, not restricted sample population. Starting with a 75-item scale, a reduced 23-item scale including three subscales, namely, *negative expectations, assimilation*, and *cognitive immunization*, resulted with the help of an EFA. The resulting questionnaire showed an excellent internal consistency and a good to excellent consistency for the three factors. Furthermore, the suggested three-factor structure of the 23-item scale could be confirmed in a second study by a CFA, showing good-fit measures. The internal consistency remained good to excellent for the overall questionnaire and the three subscales. A very good test–retest reliability could be proven.

Validity analyses showed significant correlations between the sum score of the IMS and its subscales, as well as validated questionnaires measuring depressive symptoms (BDI-II), anxiety symptoms (BAI), depressive expectations (DES), and psychological flexibility (FAH-II), indicating good concurrent validity. Consistent with the assumptions, in both studies, the IMS score was the highest correlated with the FAH-II, measuring experiential avoidance, and the DES, measuring negative expectations. As the IMS includes the subscale of negative expectations, a high correlation with the DES was expected. Experiential avoidance can be defined as “the phenomenon that occurs when a person is unwilling to remain in contact with particular private experiences (e.g., bodily sensations, emotions, thoughts, memories, images, and behavioral predispositions). It takes steps to alter the form or frequency of these experiences or the contexts that occasion them, even when these forms of avoidance cause behavioral harm” (Hayes et al., 2004). Interestingly, the IMS, especially factor 1 integrating negative expectations, is highly correlated with the FAH-II. Looking at the formulation of items of the FAH-II, they express very rigid and negative assumptions directed to the future. Moreover, as assimilation and immunization processes are also defined as avoidance processes, the high correlation was expected. This again underlines the idea of defining expectations as “core features of mental health” (Rief et al., 2015). The positive correlations with depressive and anxiety symptoms are consistent with the assumption that assimilation and immunization processes play a central role in psychopathology, but are not reflecting psychopathological symptoms *per se*. The subscales assimilation and cognitive immunization are highly correlated with the amount of negative expectations measured by the first subscale and the DES. This implies that individuals with high levels of assimilation and immunization show a higher amount of negative expectations, matching the idea of lacking expectation change leading to the persistence of expectations (Rief et al., 2015; Rief and Joormann, 2019). The comparison of people with and without a diagnosed mental disorder, as well as those having absolved psychotherapy in the past, goes in the same direction of interpretation, concluding higher assimilation and immunization processes in psychopathology.

### Research and practical implications

The ViolEx model (Rief et al., 2015) has initiated a relatively new branch of research analyzing the specific role of expectations and its adaptation mechanisms in psychopathology. However, it needs to be empirically confirmed. Until now, the ViolEx model including immunization strategies is only indirectly assessed by experimental paradigms (D’Astolfo et al., 2019; Kube et al., 2019c). In these studies, situation-specific negative expectations are induced through certain feedback. In the next step, these induced expectations are systematically violated, and the expectation change is observed. The lack of expectation change is then defined as immunization, whereas other factors could also be responsible for the expectation persistence (e.g., paradigm properties and characteristics of the induced expectations). The IMS is a promising and helpful tool to operationalize assimilation and cognitive immunization in a very efficient way in further experimental studies. The article proposes possible definitions of the concepts allowing a better understanding and communication between researchers. It should be noted that further research should be done to differentiate the variety of concepts describing similar processes (e.g., psychological or cognitive inflexibility vs. cognitive immunization). The same applies to the idea of dysfunctional versus negative expectations: There is already the literature showing that depressed people have a more realistic worldview [see optimistic bias, Sharot (2011); Korn et al. (2014)]. It fosters the idea that it is not the content of negative expectations that is the leading problem, but that the focus should rather be directed toward the processing and the handling of information. Further literature should enlighten this. In addition, the questionnaire strongly facilitates the influence of assimilation and immunization processes for various scientific questions, such as verifying the ViolEx model. The IMS enables researchers to analyze the factors responsible for expectation persistence, such as personality traits, social surroundings, or prior experiences, as proposed by the ViolEx model. Moreover, the IMS should be tested in a more severe clinical sample to ensure a valid comparison between mentally healthy and psychopathological samples.

In the context of cognitive behavioral therapy, practitioners observe the consistency of certain dysfunctional cognitions, including expectations, even if certain cognitive and/or behavioral interventions have been conducted (Rief et al., 2015; Rief and Glombiewski, 2016; Rief and Joormann, 2019). It is of great importance to reveal the mechanisms responsible for this rigidity. One approach to address this is expectation-focused psychological interventions (Rief and Glombiewski, 2016), defining expectations as core features of psychopathology. This questionnaire can provide the practitioner with important information about the general level of assimilation and cognitive immunization processes of the patient. Practitioners can adapt their therapy plan accordingly, by directly addressing the main problematic mechanism with the aim of making existing expectations more flexible. First, a more conscious observation of the patients’ expectations and second, a more flexible adaptation of personal expectations to the given environment could be the consequence. Moreover, a more active and conscious decision making is promoted (Grawe, 2000). The idea of flexibilizing cognitions in the sense of promoting a better adaptation to the environment is a very prominent idea in psychology and directly addressed by the approach of acceptance and commitment therapy (Hayes et al., 2012).

### Strengths and limitations

This article is the first to present a methodically clean validated questionnaire measuring assimilation and cognitive immunization processes. The supposed factor structure could be found through performed factor analyses with a very good fit. Moreover, reliability and validity analyses were already conducted in both studies and yielded promising results. Yet, several limitations should be considered. First, the sample of this study to develop the IMS consisted of a predominantly healthy population. In both studies, more females participated than men. Moreover, the race and ethnicity were not explicitly assessed, although an overrepresentation of the white ethnic category is assumed. Therefore, the generalization of the questionnaire is still limited (Simons et al., 2017). The IMS should be tested in clinical samples to evaluate the ability of discrimination between healthy and psychopathological groups. Due to the finding of a left-skewed distribution of the IMS and the correlations with questionnaires measuring psychopathological symptoms, a certain group discrimination can be assumed. First, comparative analyses show discriminative results, but a rather small group of individuals with psychotherapy experience and a diagnosed mental disorder was included in the studies. Second, further validity analyses should be performed to define predictive and content validity. The translated IMS should also be validated in an English-speaking population. Moreover, it would be important to find out whether certain interventions such as expectation-focused psychological interventions (Rief and Glombiewski, 2016) are able to change IMS scores.

## Conclusion

With the IMS, the first self-rating scale for the assessment of two important processes leading to rigid maintenance of expectations was developed. These processes are (a) assimilation leading to non-tested expectations and (b) cognitive immunization as a form of interpretation of certain expectation violations. In this article, the IMS showed an excellent internal consistency in two independent studies. An overall score of assimilation and/or cognitive immunization can be drawn, which will be useful in experimental research, clinical trials, and clinical practice, as it enables the direct assessment of underlying mechanisms concerning the maintenance of certain expectations.

## Data availability statement

The data that support the findings of this study are available from the corresponding author upon reasonable request.

## Ethics statement

The studies involving human participants were reviewed and approved by Ethics Committee of the Department of Psychology, Philipps-University Marburg. The patients/participants provided their written informed consent to participate in this study.

## Author contributions

A-CE was involved in conceptualization, data curation, formal analysis, investigation, project administration, resources, software, visualization, and writing—original draft. MW was involved in conceptualization, supervision, writing—review and editing, and supervision. WR was involved in supervision and funding acquisition. All authors contributed to the article and approved the submitted version.
