# Supplementary material for: Exploring the path of persisting dysfunctional expectations—Development of the immunization scale IMS
Source: Front Psychol. 2022 Dec 8;13:1033078. doi: 10.3389/fpsyg.2022.1033078 (PMC9773141; doi:10.3389/fpsyg.2022.1033078)
Supplement: Supplementary file 1 [file Data_Sheet_1.pdf]

Appendix

A1. Item-analysis of the 75-item scale

Component 1

Row	*Missings*	*Mean*	*SD*	*Skew*	*Item Difficulty*	*Item Discrimination*	*α if deleted*
NE_1	0.00 %	2.65	1.29	0.21	0.53	0.69	0.98
NE_2	0.00 %	2.56	1.21	0.35	0.51	0.68	0.98
NE_3	0.00 %	3.35	1.2	-0.38	0.67	0.61	0.98
NE_4	0.00 %	3.16	1.3	-0.17	0.63	0.59	0.98
NE_5	0.00 %	2.82	1.34	0.17	0.56	0.60	0.98
NE_6	0.00 %	2.02	1.23	0.99	0.40	0.66	0.98
NE_7	0.00 %	1.98	1.06	0.92	0.40	0.45	0.98
NE_8	0.00 %	2.09	1.12	0.86	0.42	0.54	0.98
NE_9	0.00 %	2.32	1.25	0.6	0.46	0.71	0.98
NE_10	0.00 %	2.08	1.21	0.87	0.42	0.66	0.98
NE_11	0.00 %	2.31	1.29	0.61	0.46	0.67	0.98
NE_12	0.00 %	2.52	1.33	0.35	0.50	0.60	0.98
NE_13	0.00 %	2.5	1.26	0.32	0.50	0.64	0.98
NE_14	0.00 %	3.06	1.23	-0.02	0.61	0.21	0.98
PF_1	0.00 %	2.89	1.2	-0.05	0.58	0.66	0.98
PF_2	0.00 %	2.52	1.13	0.27	0.50	0.69	0.98
PF_3	0.00 %	2.77	1.17	0.06	0.55	0.64	0.98
PF_4	0.00 %	2.72	1.2	0.21	0.54	0.56	0.98
PF_5	0.00 %	2.5	1.16	0.36	0.50	0.75	0.98
PF_6	0.00 %	2.13	1.24	0.74	0.43	0.75	0.98
PF_7	0.00 %	2.51	1.34	0.39	0.50	0.72	0.98
PF_8	0.00 %	2.66	1.2	0.01	0.53	0.49	0.98
PF_9	0.00 %	2.48	1.17	0.33	0.50	0.65	0.98
PF_10	0.00 %	2.34	1.26	0.54	0.47	0.68	0.98
PF_11	0.00 %	2.43	1.26	0.48	0.49	0.55	0.98
PF_12	0.00 %	2.55	1.23	0.22	0.51	0.59	0.98
PF_13	0.00 %	2.38	1.23	0.35	0.48	0.72	0.98
PF_14	0.00 %	2.1	1.19	0.83	0.42	0.53	0.98
PF_15	0.00 %	2.43	1.27	0.42	0.49	0.76	0.98
PF_16	0.00 %	2.71	1.31	0.13	0.54	0.74	0.98
PF_17	0.00 %	2.68	1.26	0.16	0.54	0.69	0.98
PF_18	0.00 %	2.57	1.13	0.09	0.51	0.25	0.98
PF_19	0.00 %	2.46	1.25	0.31	0.49	0.69	0.98
PF_20	0.00 %	2.49	1.15	0.15	0.50	0.71	0.98
A_1	0.00 %	1.97	1.1	0.98	0.39	0.71	0.98
A_2	0.00 %	2.39	1.21	0.43	0.48	0.64	0.98
A_3	0.00 %	2.24	1.19	0.6	0.45	0.67	0.98
A_4	0.00 %	2.55	1.23	0.34	0.51	0.72	0.98
A_5	0.00 %	2.77	1.27	0.08	0.55	0.54	0.98
A_6	0.00 %	2	1.08	0.77	0.40	0.66	0.98
A_7	0.00 %	2.3	1.16	0.38	0.46	0.55	0.98
A_8	0.00 %	2.32	1.23	0.66	0.46	0.54	0.98
A_9	0.00 %	2.53	1.14	0.23	0.51	0.74	0.98
A_10	0.00 %	2.63	1.29	0.2	0.53	0.50	0.98
A_11	0.00 %	2.5	1.28	0.41	0.50	0.65	0.98
A_12	0.00 %	2.63	1.2	0.09	0.53	0.44	0.98
A_13	0.00 %	2.85	1.32	0.08	0.57	0.62	0.98
A_14	0.00 %	2.97	1.25	-0.06	0.59	0.67	0.98
A_15	0.00 %	2.78	1.23	0.12	0.56	0.69	0.98
CIM_1	0.00 %	2.32	1.18	0.61	0.46	0.70	0.98
CIM_2	0.00 %	2.08	1.06	0.87	0.42	0.70	0.98
CIM_3	0.00 %	2.25	1.13	0.56	0.45	0.62	0.98
CIM_4	0.00 %	2.26	1.21	0.55	0.45	0.71	0.98
CIM_5	0.00 %	2.22	1.2	0.53	0.44	0.69	0.98
CIM_6	0.00 %	2.23	1.22	0.64	0.45	0.51	0.98
CIM_7	0.00 %	2.42	1.25	0.55	0.48	0.64	0.98
CIM_8	0.00 %	2.12	1.08	0.7	0.42	0.64	0.98
CIM_9	0.00 %	2.47	1.3	0.28	0.49	0.71	0.98
CIM_10	0.00 %	1.83	1.04	1.13	0.37	0.54	0.98
CIM_11	0.00 %	1.68	0.96	1.45	0.34	0.44	0.98
CIM_12	0.00 %	1.78	1.03	1.2	0.36	0.51	0.98
CIM_13	0.00 %	2.17	1.19	0.61	0.43	0.63	0.98
CIM_14	0.00 %	2.13	1.11	0.65	0.43	0.69	0.98
CIM_15	0.00 %	2.13	1.17	0.65	0.43	0.60	0.98
CIM_16	0.00 %	2.41	1.28	0.39	0.48	0.71	0.98
CIM_17	0.00 %	2.31	1.21	0.47	0.46	0.72	0.98
CIM_18	0.00 %	2.37	1.28	0.44	0.47	0.70	0.98
CIM_19	0.00 %	2.41	1.16	0.37	0.48	0.67	0.98
CIM_20	0.00 %	2.19	1.15	0.65	0.44	0.65	0.98
CIM_21	0.00 %	2.34	1.23	0.48	0.47	0.70	0.98
CIM_22	0.00 %	2.53	1.29	0.27	0.51	0.72	0.98
CIM_23	0.00 %	2.43	1.17	0.27	0.49	0.68	0.98
CIM_24	0.00 %	2.06	1.11	0.7	0.41	0.68	0.98
CIM_25	0.00 %	1.98	1.07	0.73	0.40	0.62	0.98
CIM_26	0.00 %	1.92	1.12	0.93	0.38	0.59	0.98

Mean inter-item-correlation=0.405 · Cronbach's α=0.981

A2. Factor loadings of 73-item-scale

	WLS2	WLS1	WLS3	WLS4
IM04_01	0.25	0.72	0.12	0.24
IM04_02	0.31	0.66	0.13	0.20
IM04_03	0.12	0.65	0.24	0.16
IM04_04	0.16	0.63	0.25	
IM04_05	0.19	0.66	0.17	0.13
IM04_06	0.40	0.64		0.10
IM04_07	0.32	0.34	0.14	
IM04_08	0.37	0.39	0.21	
IM04_09	0.35	0.68	0.15	0.18
IM04_10	0.30	0.70		0.17
IM04_11	0.22	0.76	0.12	0.19
IM04_12	0.30	0.57	0.17	0.11
IM04_13	0.17	0.56	0.28	0.27
IM05_01	0.15	0.54	0.26	0.38
IM05_03	0.25	0.46	0.22	0.49
IM05_04		0.45	0.39	0.42
IM05_05		0.48	0.36	0.18
IM05_06	0.19	0.65	0.36	0.28
IM05_07	0.29	0.67	0.27	0.26
IM05_08	0.22	0.68	0.24	0.26
IM05_09		0.36	0.42	0.12
IM05_10	0.19	0.46	0.48	0.16
IM05_11	0.27	0.53	0.35	0.17
IM05_12	0.24	0.39	0.36	
IM05_13	0.11	0.44	0.46	0.19
IM05_14	0.37	0.29	0.27	0.59
IM05_15	0.19	0.44	0.23	0.19
IM05_16	0.26	0.44	0.29	0.61
IM05_17	0.17	0.46	0.40	0.52
IM05_18	0.11	0.43	0.42	0.50
IM05_20	0.29	0.45	0.25	0.42
IM05_21	0.28	0.39	0.36	0.45
IM06_01	0.49	0.30	0.42	0.22
IM06_02	0.35	0.19	0.61	0.16
IM06_03	0.47	0.25	0.53	
IM06_04	0.38	0.46	0.42	0.16
IM06_05	0.42	0.34	0.32	
IM06_06	0.53	0.32	0.32	0.11
IM06_07	0.39	0.13	0.41	0.19
IM06_08	0.26	0.14	0.53	0.21
IM06_09	0.37	0.30	0.40	0.48
IM06_10	0.13	0.15	0.57	0.24
IM06_11	0.29	0.24	0.62	0.21
IM06_12	0.13	0.15	0.54	0.10
IM06_13	0.24	0.28	0.62	0.14
IM06_14	0.29	0.27	0.62	0.22
IM06_15	0.33	0.30	0.55	0.24
IM07_01	0.43	0.26	0.19	0.62
IM07_02	0.42	0.22	0.28	0.56
IM07_03	0.48	0.20	0.32	0.26
IM07_04	0.56	0.16	0.19	0.59
IM07_05	0.52	0.21	0.13	0.57
IM07_06	0.56		0.14	0.22
IM07_07	0.60	0.29	0.11	0.25
IM07_08	0.68	0.13	0.18	0.28
IM07_09	0.59	0.23	0.12	0.51
IM07_10	0.49	0.13	0.24	0.25
IM07_11	0.39	0.23	0.12	
IM07_12	0.52	0.22	0.11	0.13
IM07_13	0.67	0.13	0.21	0.24
IM07_14	0.65	0.15	0.32	0.26
IM07_15	0.61	0.13	0.26	0.19
IM07_16	0.41	0.19	0.39	0.52
IM07_17	0.41	0.32	0.30	0.47
IM07_18	0.41	0.29	0.23	0.54
IM08_01	0.68	0.35	0.11	0.12
IM08_02	0.66	0.26	0.21	0.12
IM08_03	0.72	0.36		0.19
IM08_04	0.50	0.38	0.15	0.43
IM08_05	0.68	0.33	0.19	
IM08_06	0.61	0.22	0.26	0.27
IM08_07	0.71	0.13	0.17	0.21
IM08_08	0.57	0.30	0.21	

	WLS2	WLS1	WLS3	WLS4
SS loadings	12.20	12.06	7.79	6.90
Proportion Var	0.17	0.17	0.11	0.09
Cumulative Var	0.17	0.33	0.44	0.53

A3. Items of the Immunization Scale (IMS) – English Version

	**Do not agree**	**Do Rather not agree**	**neutral**	**Rather agree**	**agree**
**Negative expectations**					
I rarely expected good things to happen.					
I often worried about future events.					
I generally had many negative expectations.					
I often expected to be left alone with my problems.					
I expected not to be able to deal well with my feelings.					
Negative expectations made my life difficult.					
**Assimilation**					
If I had a negative expectation, …					
... I was rarely curious about what would happen.					
...I did not like being surprised.					
... I tried not to think about the expectation.					
... it made me avoid certain situations or people.					
... it was difficult to be open to the situation or experience.					
… it largely controlled my behavior.					
**Immunisation**					
If I had an experience that did not correspond with my negative expectation, then ...					
...I still held on to that expectation.					
...it was usually an exception.					
...something was wrong.					
...I usually found an explanation why the expectation was still right.					
If something went well, although I had a negative expectation, then...					
...it was just luck or coincidence.					
...I could not be responsible for it.					
...this was an exception.					
...it was only due to the specific situation.					
...there was no point in questioning the expectation anyway.					
...my expectation was still right.					
...it was just fate.					

Items of the Immunization Scale (IMS) – German Version

	Stimme nicht zu	Stimme eher nicht zu	Teils/teils	Stimme eher zu	Stimme zu
**Negative Erwartungen**					
Ich ging selten vom Guten aus.					
Ich machte mir oft Sorgen über zukünftige Ereignisse.					
Ich hatte im Allgemeinen viele negative Erwartungen.					
Ich erwartete häufig allein mit meinen Problemen gelassen zu werden.					
Ich erwartete, nicht gut mit meinen Gefühlen umgehen zu können.					
Negative Erwartungen machten mir das Leben schwer.					
**Vermeidung**					
Wenn ich eine negative Erwartung hatte, …					
...war ich selten neugierig, was passieren wird.					
...ließ ich mich nicht gerne überraschen.					
...versuchte ich nicht über die Erwartung nachzudenken.					
...führte diese dazu, dass ich verschiedene Situationen oder Personen vermied.					
...war es schwierig dem Ereignis oder der Erfahrung offen gegenüberzutreten.					
...steuerte diese Erwartung größtenteils mein Verhalten.					
**Immunisierung**					
Wenn ich eine Erfahrung machte, die nicht meiner negativen Erwartung entsprach, …					
...hielt ich trotzdem an dieser Erwartung fest.					
...war das meistens eine Ausnahme.					
...stimmte irgendetwas nicht.					
...fand ich meistens eine Erklärung, wieso die Erwartung trotzdem richtig war.					
Wenn mal etwas gut gelaufen ist, obwohl ich eine negative Erwartung hatte…					
...war das nur Glück oder Zufall.					
...konnte ich nicht dafür verantwortlich sein.					
...war das eine Ausnahme.					
...lag das nur an der spezifischen Situation.					
...brachte es sowieso nichts, die Erwartung in Frage zu stellen.					
...war meine Erwartung trotzdem richtig.					
...war es nur Schicksal.					
